# Supplementary material for: Rapid turnover of life-cycle-related genes in the brown algae
Source: Genome Biol. 2019 Feb 14;20:35. doi: 10.1186/s13059-019-1630-6 (PMC6374913; doi:10.1186/s13059-019-1630-6)
Supplement: Supplementary file 3 — Protein alignments, corresponding Gblocks html file and CDS sequences for the lineage conserved gametophyte-biased genes. (ZIP 2109 kb) [file 13059_2019_1630_MOESM3_ESM.zip › Con_GA_alignments/Ec-21_000910.aa_ali.fasta-gb.htm]

Ec-21\_000910.aa.fasta.trans.aa\_ali.fasta


## Gblocks 0.91b Results

Processed file: **Ec-21\_000910.aa.fasta.trans.aa\_ali.fasta**  
Number of sequences: **2**  
Alignment assumed to be: **Protein**  
New number of positions: **388** (selected positions are underlined in blue)

```
                         10        20        30        40        50        60
                 =========+=========+=========+=========+=========+=========+
Ec-21_000910     MLTSNRLSVVDARAIVGLPAVRGSSSSTTDLSPLDQHRASPAHLDGSVPLLGREFGGTLK
MSTRG.17132.1    ------------------------------------------------------------
                                                                             


                         70        80        90       100       110       120
                 =========+=========+=========+=========+=========+=========+
Ec-21_000910     SRFASLARKSGADRKETRDHGRSLVGRRANLEGRLGDDGDPGRVSAGTQEPLVSPLQRFQ
MSTRG.17132.1    ------------------------------------------------------------
                                                                             


                        130       140       150       160       170       180
                 =========+=========+=========+=========+=========+=========+
Ec-21_000910     KAGRKIVRQGLQHRLQATPDDRKLLGRLGSLEVADGDLENAIEHLRLAAKGSDDDVAWTT
MSTRG.17132.1    ------------------------------------------------------------
                                                                             


                        190       200       210       220       230       240
                 =========+=========+=========+=========+=========+=========+
Ec-21_000910     LARAEVRAWEQDPATGHERLRWACDAYRRSVDLLEPGFVQVWELPARLLELGGVYESFGS
MSTRG.17132.1    ------------------------------------------------------------
                                                                             


                        250       260       270       280       290       300
                 =========+=========+=========+=========+=========+=========+
Ec-21_000910     FEGALHIYQRIASSMPLSSGFEGVLFRCAVVMRYMATLERAPRNGLLESALEHLDLIMQE
MSTRG.17132.1    ------------------------------------------------------------
                                                                             


                        310       320       330       340       350       360
                 =========+=========+=========+=========+=========+=========+
Ec-21_000910     QALRKGFFAESAALLYADVCMALAEGSAVAKGWAKASYQEVFDNRKAQGDKHAMTFRDWD
MSTRG.17132.1    ------------------------------------------------------------
                                                                             


                        370       380       390       400       410       420
                 =========+=========+=========+=========+=========+=========+
Ec-21_000910     DWIASPETQLQLSKEWSQKGEPALAVLAFEKALEAFGRQKEISPSSKAEPSFSALMDASE
MSTRG.17132.1    -------------------------------------------------------MDASE
                                                                        #####


                        430       440       450       460       470       480
                 =========+=========+=========+=========+=========+=========+
Ec-21_000910     AYASFQRFVRAEEVVRKALTLQPENEEAKGLLRELVGDENCRRKEMGLQVVLHLQSKWRT
MSTRG.17132.1    AWASFQRFSRAETMVRKALLLEPESEEAKDLLKELVGNEDCRRKEMRLQVALHVQSKWRT
                 ############################################################


                        490       500       510       520       530       540
                 =========+=========+=========+=========+=========+=========+
Ec-21_000910     RVWQGEYLDSLKTCLRRNLEERLAKNRLDMEARDLLAYFFQEKHRPVVLYEDRCARVIQR
MSTRG.17132.1    RVWQAGYLRSLKSNLKICLEGRLENNRMEIDTREQLSYFFREKHRPVLLYEDRCARIIQR
                 ############################################################


                        550       560       570       580       590       600
                 =========+=========+=========+=========+=========+=========+
Ec-21_000910     LGRAGLFRLRWFAAKKAAYRTQLTNALKLWQKSGYNAESRAIIRARASSRYTPRTHKIVS
MSTRG.17132.1    LGRAGLFRLRWFAVKRKTYRKQLTSALKLWQKDGYEAESREIIRSRASNRYTPSTHKIVS
                 ############################################################


                        610       620       630       640       650       660
                 =========+=========+=========+=========+=========+=========+
Ec-21_000910     VVALMDSQEEAAPLLQRAARSMLARKMFRKVLKNRARDMRRQRKAAAIRLQCQLRRFFAR
MSTRG.17132.1    VVALMDRQDRAIPFIQRAARSLLARKMMLKILKSRTKDLKRKRRAAAVHLQCQVRGYLAR
                 ############################################################


                        670       680       690       700       710       720
                 =========+=========+=========+=========+=========+=========+
Ec-21_000910     REVDRRISHAAQRIRAATFIQRVYRARALQWSYAVKRAQLARERKIAKAKLGSSIKLQAT
MSTRG.17132.1    RELDRRVALVAARTRAATSIQTVYRDRALLWEFAVKRAQYAHERDVAKARFVSARKLQAT
                 ############################################################


                        730       740       750       760       770       780
                 =========+=========+=========+=========+=========+=========+
Ec-21_000910     WRMKQARYEVVVRRRAVSDIQRSTRGFLGRRRAAERKHALVTRIQRQYRRHRERMVILTA
MSTRG.17132.1    WRMKRGYITVADRRRAVLDIQRSFRGFLGRKRSERRKKELVSRIQRQYRRHRERLVIVQA
                 ############################################################


                        790       800       810       820       830       840
                 =========+=========+=========+=========+=========+=========+
Ec-21_000910     LLAMRASREKKEERAREVERRFRALEEQRLGNTSGSEAIATPLDKRRAERAHKRLLRRIK
MSTRG.17132.1    LLAIRISRDHKEARAKEIERRFR-------------------------------------
                 #######################                                     


                        850       860       870       880       890       900
                 =========+=========+=========+=========+=========+=========+
Ec-21_000910     AKGSSVVRARRLALQRAGKPSSPSQPTRGIPPTRSLSPPTSRRASAFSTAPLLQSDTCNE
MSTRG.17132.1    ------------------------------------------------------------
                                                                             


                        910       920       930       940       950       960
                 =========+=========+=========+=========+=========+=========+
Ec-21_000910     SNRRQSIPDQEEEGIRRISPTGAHVTHQSAAAVLSAEVACSSSAPGASALPAGHETLLSG
MSTRG.17132.1    ------------------------------------------------------------
                                                                             


                        970       980       990      1000      1010      1020
                 =========+=========+=========+=========+=========+=========+
Ec-21_000910     GSTPRLVEDATSSPSSHPSTTTAKPTSQRPSGGAAIKSKTGACGWSPSRTKLQRSQTSPP
MSTRG.17132.1    ------------------------------------------------------------
                                                                             


                       1030      1040      1050      1060      1070      1080
                 =========+=========+=========+=========+=========+=========+
Ec-21_000910     PPSSPALKAALKCARGVGGRLGTPLGSPFPAPGEDWAAGRAASGAASAAARVSVKLSGRS
MSTRG.17132.1    ------------------------------------------------------------
                                                                             


                       1090      1100      1110      1120      1130      1140
                 =========+=========+=========+=========+=========+=========+
Ec-21_000910     FEPSAPGSRFFPPFGPTGAASDGALCAPVVFEMVQDAADDEEDADGADNDVAGRVRGWHG
MSTRG.17132.1    ------------------------------------------------------------
                                                                             


                       1150      1160      1170      1180      1190      1200
                 =========+=========+=========+=========+=========+=========+
Ec-21_000910     LTLYRSPGVDQRSPVFQQALQLETIAFSPDVQTPPRSTPPQSPCRGSGSSRKYDASGERP
MSTRG.17132.1    ------------------------------------------------------------
                                                                             


                       1210      1220      1230      1240      1250      1260
                 =========+=========+=========+=========+=========+=========+
Ec-21_000910     QPATSICHGQSASWRALACGDQERKGLGMSSSPERGAGRQKGRTDDASKPRPRPWDELES
MSTRG.17132.1    ------------------------------------------------------------
                                                                             


                       1270      1280      1290      1300      1310      1320
                 =========+=========+=========+=========+=========+=========+
Ec-21_000910     LEHTRREEREQEQQVRDVGGTYFATPELHHYGVVKSIPSPPQFTTTKPDGAMSVTTAPLH
MSTRG.17132.1    ------------------------------------------------------------
                                                                             


                       1330      1340      1350      1360      1370      1380
                 =========+=========+=========+=========+=========+=========+
Ec-21_000910     VASEASTLFDSGDMIMVAALLAHPECSLKGFVLHRAKLDTPPGREALLKCLELNKLSRLA
MSTRG.17132.1    ------------------------------------------------------------
                                                                             


                       1390      1400      1410      1420      1430      1440
                 =========+=========+=========+=========+=========+=========+
Ec-21_000910     LGGCVWFDDAMRTDNSRQYEWRTGRWWEKVCDTIRTSAFRLREVVVEGLERGGDALGTGI
MSTRG.17132.1    ------------------------------------------------------------
                                                                             


                       1450      1460      1470      1480      1490      1500
                 =========+=========+=========+=========+=========+=========+
Ec-21_000910     GTVLGDYFAKRFGSLASISLVRCGLSDEGATAVARGIWASSGLTHVDLSANAISDVGVEA
MSTRG.17132.1    ------------------------------------------------------------
                                                                             


                       1510      1520      1530      1540      1550      1560
                 =========+=========+=========+=========+=========+=========+
Ec-21_000910     LAQALVGDYPLDLDEGEEGASGSHEEPFRSHRPPLIKTLDLGQNRITDPGGTTLGAALPR
MSTRG.17132.1    ------------------------------------------------------------
                                                                             


                       1570      1580      1590      1600      1610      1620
                 =========+=========+=========+=========+=========+=========+
Ec-21_000910     SVFLESITLSYNFMMEEAAQALLSAAASSRPTLKEIHCEGNLFDEDVCGHIAQALKRRDE
MSTRG.17132.1    ------------------------------------------------------------
                                                                             


                       1630      1640      1650      1660      1670      1680
                 =========+=========+=========+=========+=========+=========+
Ec-21_000910     RISPPRKPRRDVPGITSPTPTGQIRTGNDWFDCSPNDALACLRGGERNLARIDVGGSRVP
MSTRG.17132.1    ------------------------------------------------------------
                                                                             


                       1690      1700      1710      1720      1730      1740
                 =========+=========+=========+=========+=========+=========+
Ec-21_000910     ARTPKVGRSLPGDGRVTGDNSVTFPTVADCRSRKRGHDQAQEQRDEREAVSSSYLAAAIG
MSTRG.17132.1    ------------------------------------------------------------
                                                                             


                       1750      1760      1770      1780      1790      1800
                 =========+=========+=========+=========+=========+=========+
Ec-21_000910     CPRIPAAAVGAVGSKEETGRLPLDTDQRHRHGEQPSYNPMGNQFREGLELGQTRFRQAAR
MSTRG.17132.1    ------------------------------------------------------------
                                                                             


                 
                 ==
Ec-21_000910     IV
MSTRG.17132.1    --
```

```
Parameters used
Minimum Number Of Sequences For A Conserved Position: 2
Minimum Number Of Sequences For A Flanking Position: 2
Maximum Number Of Contiguous Nonconserved Positions: 8
Minimum Length Of A Block: 10
Allowed Gap Positions: None
Use Similarity Matrices: Yes
```

```
Flank positions of the 1 selected block(s)
Flanks: [416  803]  

New number of positions in Ec-21_000910.aa.fasta.trans.aa_ali.fasta-gb:  388  (21% of the original 1802 positions)
```
